# Caspase‐8 regulates the expression of pro‐ and anti‐inflammatory cytokines in human bone marrow‐derived mesenchymal stromal cells

**DOI:** 10.1002/iid3.117

**Published:** 2016-07-21

**Authors:** Siv H. Moen, Marita Westhrin, Muhammad Zahoor, Nikolai N. Nørgaard, Hanne Hella, Berit Størdal, Anders Sundan, Nadra J. Nilsen, Anne‐Marit Sponaas, Therese Standal

**Affiliations:** ^1^ The KG Jebsen Center for Myeloma Research and Faculty of Medicine, Department of Cancer Research and Molecular MedicineNorwegian University of Science and Technology (NTNU)TrondheimNorway; ^2^ Centre of Molecular Inflammation Rearch (CEMIR)NTNUTrondheimNorway

**Keywords:** Caspase‐8, cytokines, human bone marrow‐derived MSCs (hBMSCs)

## Abstract

**Introduction:**

Mesenchymal stem cells, also called mesenchymal stromal cells, MSCs, have great potential in stem cell therapy partly due to their immunosuppressive properties. How these cells respond to chronic inflammatory stimuli is therefore of importance. Toll‐like receptors (TLR)s are innate immune receptors that mediate inflammatory signals in response to infection, stress, and damage. Caspase‐8 is involved in activation of NF‐kB downstream of TLRs in immune cells. Here we investigated the role of caspase‐8 in regulating TLR‐induced cytokine production from human bone marrow‐derived mesenchymal stromal cells (hBMSCs).

**Methods:**

Cytokine expression in hBMCs in response to poly(I:C) and LPS was evaluated by PCR, multiplex cytokine assay, and ELISA. TLR3, TRIF, and caspase‐8 were silenced using siRNA. Caspase‐8 was also inhibited using a caspase‐8 inhibitor, z‐IEDT.

**Results:**

We found that TLR3 agonist poly(I:C) and TLR4 agonist LPS induced secretion of several pro‐inflammatory cytokines in a TLR‐dependent manner which required the TLR signaling adaptor molecule TRIF. Further, poly(I:C) reduced the expression of anti‐inflammatory cytokines HGF and TGFβ whereas LPS reduced HGF expression only. Notably, caspase‐8 was involved in the induction of IL‐ IL‐1β, IL‐6, CXCL10, and in the inhibition of HGF and TGFβ.

**Conclusion:**

Caspase‐8 appears to modulate hBMSCs into gaining a pro‐inflammatory phenotype. Therefore, inhibiting caspase‐8 in hBMSCs might promote an immunosuppressive phenotype which could be useful in clinical applications to treat inflammatory disorders.

## Introduction

Mesenchymal stem cells, also known as mesenchymal stromal cells (MSCs), have regenerative and immunosuppressive properties, and are therefore used in clinical trials to prevent or treat conditions like graft‐versus‐host disease, Chron's disease and multiple‐sclerosis 1, 2. MSCs can inhibit the maturation of dendritic cells, inhibit T‐cell activation and proliferation, and promote expansion of suppressive regulatory T‐cells and regulatory macrophages 3, 4, 5, 6, 7, 8. The inhibitory effect on immune cells is partly due to cell–cell contact 9, and partly due to the secretion of suppressive factors like hepatocyte growth factor (HGF), transforming growth factor β (TGFβ), IL‐10, indoleamine 2,3‐dioxygenase (IDO), and prostaglandin E2 (PGE2) by the BMSCs 3, 6, 8, 10. Tissue damage or infections can, however, change the phenotype of the MSCs from immune suppressive to immune stimulatory, and toll‐like receptors (TLRs) might play an important role in this 11, 12.

Toll like receptors (TLRs) are essential in initiating an immune response to pathogens, and can also recognize endogenously‐derived danger associated molecules (DAMPs) 13, 14. Human bone marrow‐derived MSCs (hBMSCs) express TLR1‐6 and TLR9 11, 15, 16, and TLR activation induce pro‐inflammatory cytokines such as IL‐6, CXCL8, and CXCL10 9, 11, 15. How TLR‐signaling influence the immunomodulating properties of BMSCs is unclear. For example, one study reported decreased T‐cell suppression in response to TLR3 and TLR4 activation, possibly due to reduced Jagged‐1 expression in the BMSCs 9. Another study showed increased T‐cell suppression caused by increased IDO expression by the BMSCs in response to the same ligands 17. Such differences can be explained by different experimental setups in terms of responder cells, timing and concentrations of the TLR agonists.

Signaling downstream of TLRs is initiated by adaptor molecules that contain Toll‐IL1 receptor (TIR) domains. All TLRs, except TLR3, interact with the adaptor MYD88. MYD88 recruits members of the IRAK (IL‐1 receptor‐associated kinase) family of serine‐threonine kinases, initiating a signaling cascade leading to activation of the transcription factor NF‐kB and production of pro‐inflammatory cytokines. TLR3, which recognizes dsRNA, signals only via the adaptor TRIF (TIR‐domain‐containing adapter‐inducing interferon‐β), which ultimately leads to activation of the transcription factor interferon regulatory factor 3 (IRF3) and NF‐kB. TLR4 can signal via both the MyD88‐ and the TRIF‐dependent pathway. The TRIF pathway leads to type I interferon production (IFN‐α and IFN‐β), as well as the production of pro‐inflammatory cytokines 18. Interestingly, the pro‐apoptotic caspase, caspase‐8 is involved in activation of NF‐kB downstream of TLRs 19, 20, 21, 22, 23, 24. Caspase‐8 appears to be important for both TRIF and MYD88 dependent signaling although the molecular mechanism of this is not fully clarified 25, 26. Most studies of the role of caspase‐8 in TLR signaling have been performed on myeloid cells. Whether caspase‐8 is of any importance for BMSC survival and cytokine production in response to TLR agonists is unknown.

In this study, we aimed to characterize the role of caspase‐8 for TLR‐induced cytokine production in hBMSCs. We found that the dsRNA mimic poly(I:C) (TLR3 ligand) and LPS (TLR4 ligand) triggered the secretion of a wide range of cytokines, including pro‐inflammatory IL‐6, CXCL10 and IL‐1β, but impaired the induction of the anti‐inflammatory cytokines HGF and TGFβ. Caspase‐8 was identified as an important mediator of TLR‐TRIF induced cytokine production in hBMSCs. In contrast, the anti‐inflammatory cytokines TGFβ and HGF were elevated in the absence of caspase‐8. Knocking down caspase‐8 did not influence survival of the hBMSCs. Our results suggest that targeting caspase‐8 in hBMSCs might promote an immunosuppressive phenotype which could be useful in clinical applications to treat inflammatory disorders.

## Results

### Poly(I:C)‐induced IL‐1β, IL‐6, and CXCL10 is TLR3‐dependent

**Figure 1:**
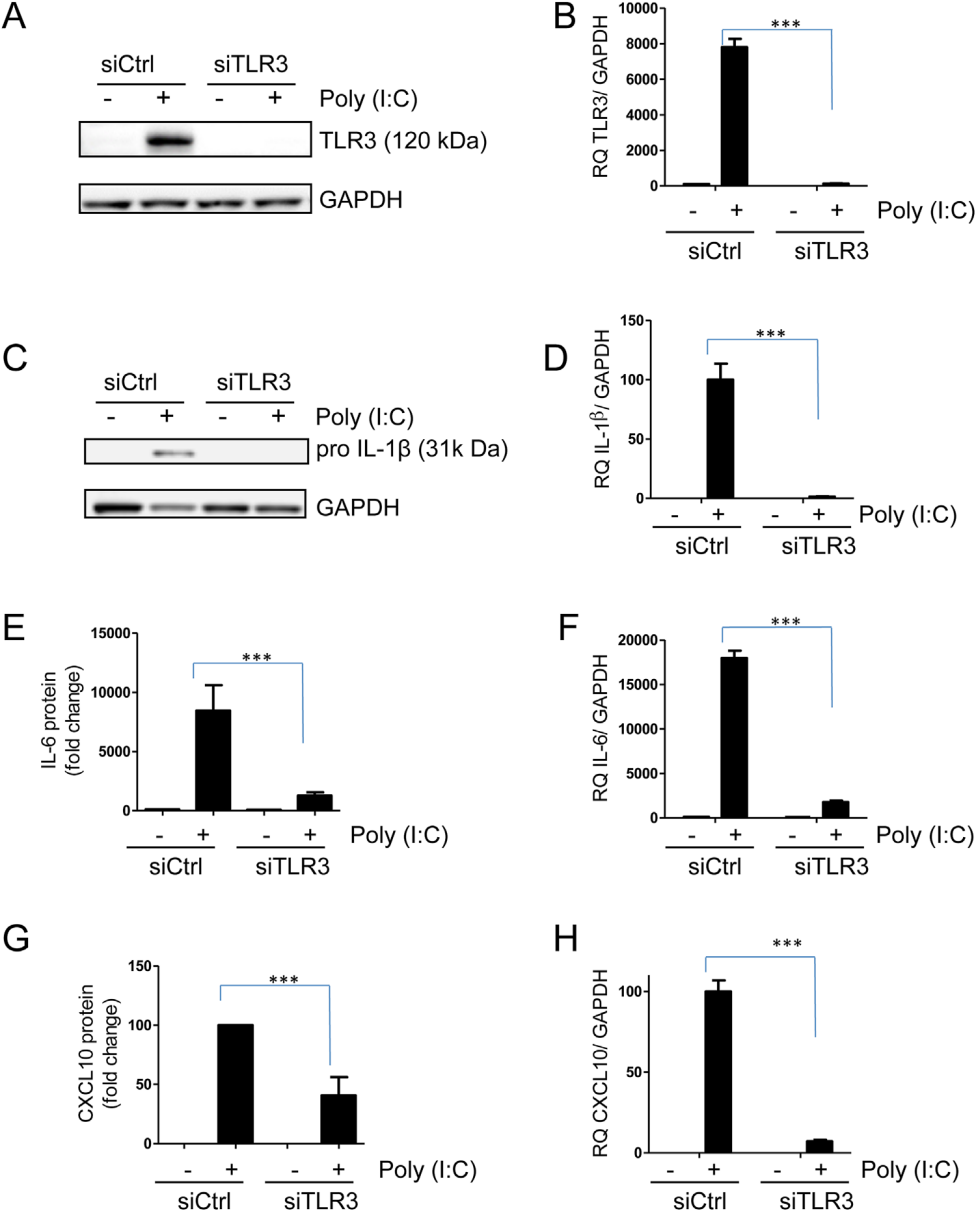
IL‐1β, IL‐6, and CXCL10 expression in response to poly(I:C). hBMSCs were transfected with non‐coding siRNA (siCtrl) or siRNA against TLR3 (siTLR3). After 48 h the cells were treated with poly(I:C) (5 µg/ml) for 24 h. Knock‐down of TLR3 was confirmed by (A) western blot and (B) QPCR. The effect of silencing TLR3 on IL‐1β mRNA was analyzed by (C) western blot and (D) QPCR. (E) IL‐6‐ and (G) CXCL10‐ protein levels in the cell culture supernatants were quantified by ELISA and presented as mean + SEM of relative protein concentrations of five independent experiments using hBMSCs obtained from three donors. Protein concentrations in untreated cells were used as reference for IL‐6 and varied from 100 to 500 pg/ml between experiments. For CXCL10 poly(I:C) stimulated cells were used as a reference and varied from 600 to 6000 pg/ml between experiments. Levels of (F) IL‐6 mRNA and (H) CXCL10 mRNA were assessed by QPCR and are plotted as mean + SD of three replicates from one representative experiment out of 3–5 using hBMSCs obtained from three donors. Data are presented as relative to (F) untreated cells or to (H) poly(I:C) treated cells. *P < 0.05, **P < 0.01, ***P ≤ 0.001 (Two‐way ANOVA/Bonferroni post test).

TLR agonists induce cytokine production in hBMSCs, in particular in response to TLR3 (poly(I:C)) and TLR4 (LPS) agonists 11, 15 (Supplementary Table S1). Poly(I:C)‐induced cytokine production can be mediated by TLR3 or by the cytosolic RNA helicases RIG‐I (retinoic acid‐inducible gene I) and MDA‐5 (melanoma differentiation‐associated gene 5) 27, 28. To test whether the cytokine production was mediated by TLR3 signaling we knocked down TLR3 in hBMSCs by siRNA. Poly(I:C) treatment for 24 h led to a marked increase in TLR3 protein and mRNA, which was inhibited in cells treated with siRNA towards TLR3 (Fig. 1A and B). Moreover, knocking down TLR3 reduced IL‐1β protein and mRNA (Fig. 1C and D). We could however not detect the mature form of IL‐1β on western blot, suggesting that TLR3 activation increased pro‐IL‐1β rather than cleavage into mature IL‐1β. Similarly, IL‐6 was reduced at both protein and mRNA level when TLR3 was silenced (Fig. 1E and F), as were CXCL10 protein and mRNA upon TLR3 knock‐down (Fig. 1G and H). These results indicate that the induction of inflammatory cytokines induced by poly(I:C) in the hBMSCs is mediated by TLR3, as also supported by previous studies 29, 30.

### LPS‐induced IL‐1β and CXCL10 is dependent on TRIF

**Figure 2:**
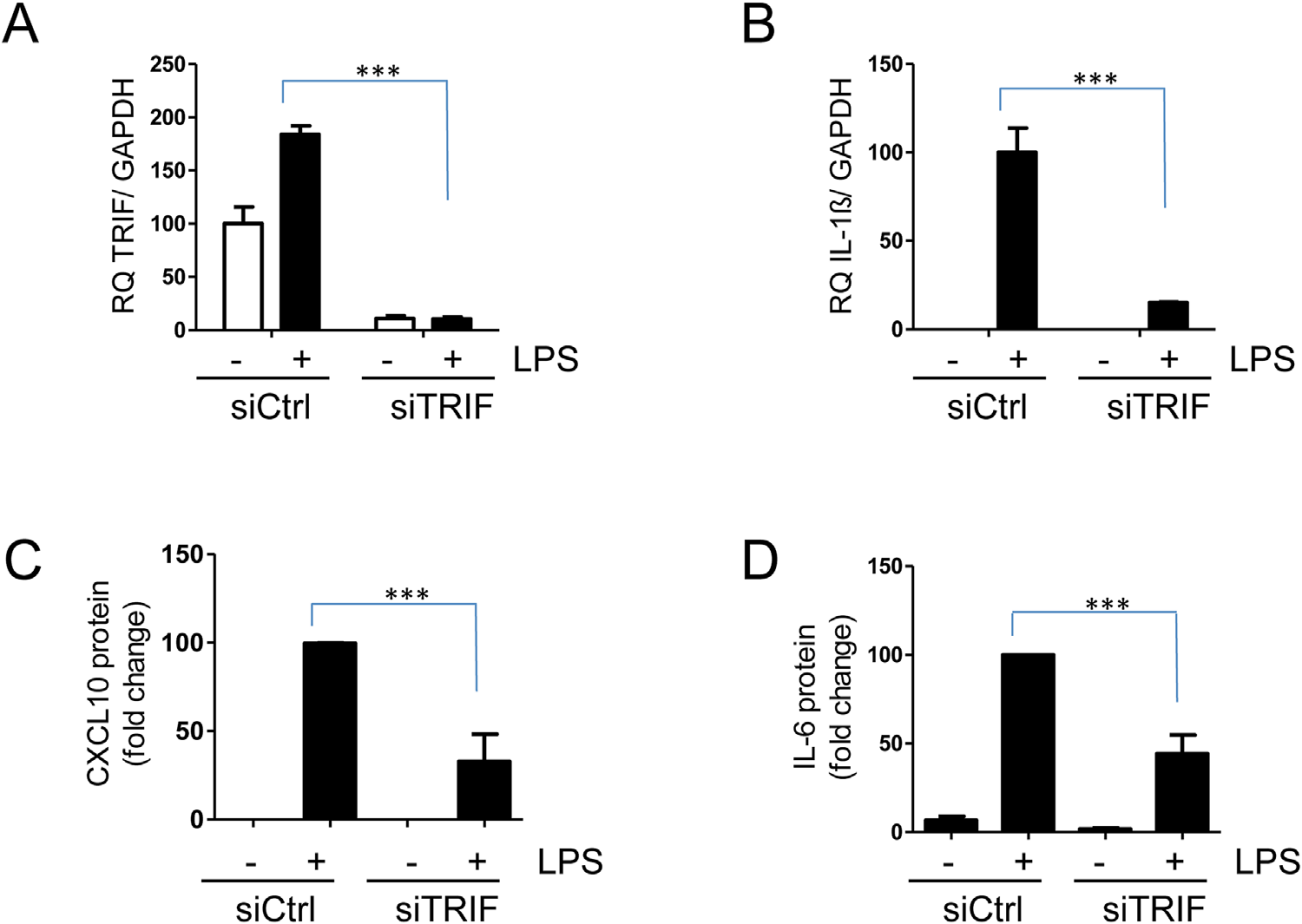
IL‐1β and CXCL10 production in response to LPS. hBMSCs were transfected with non‐coding siRNA (siCtrl) or siRNA against TRIF (siTRIF), and after 48 h the cells were treated with LPS (1 µg/ml) for another 24 h. (A) Knock‐down of TRIF was confirmed by QPCR. (B) IL‐1β mRNA was assessed by QPCR. (C) CXCL10 and (D) IL‐6 protein levels were quantified by ELISA. Data are plotted in (A and B) as mean + SD of three replicates from one representative experiment out of three using hBMSCs obtained from two different donors and in (C and D) as mean + SEM of relative protein concentrations using LPS treated cells as a reference (*n* = 3–5 independent experiments using hBMSCs obtained from four different donors). IL‐6 concentrations in LPS treated cells varied from 3500 to 15000 pg/ml between experiments, and CXCL10 concentrations in LPS treated cells varied from 600 to 6000 pg/ml between experiments. *P < 0.05, **P < 0.01, ***P ≤ 0.001 (Two‐way ANOVA/Bonferroni post test).

LPS is a well characterized ligand for TLR4. In contrast to TLR3, which signals solely via the signaling adaptor TRIF, TLR4 can signal through both MYD88‐ and the TRIF‐pathway 18. In order to determine whether the cytokine expression in response to LPS was dependent on TRIF, we knocked‐down TRIF by siRNA. When TRIF mRNA was reduced by 95% (Fig. 2A), LPS‐induced IL‐1β mRNA was reduced by 85% (Fig. 2B). Further, LPS‐induced CXCL10 protein production (Fig. 2C) and IL‐6 secretion (Fig. 2D) was significantly reduced when TRIF was knocked down. Taken together, these results show that LPS induced IL‐1β, CXCL10, and IL‐6 production is, at least partly, TRIF‐dependent.

### Production of IL‐1β, CXCL10, and IL‐6 is partly dependent on caspase‐8

**Figure 3:**
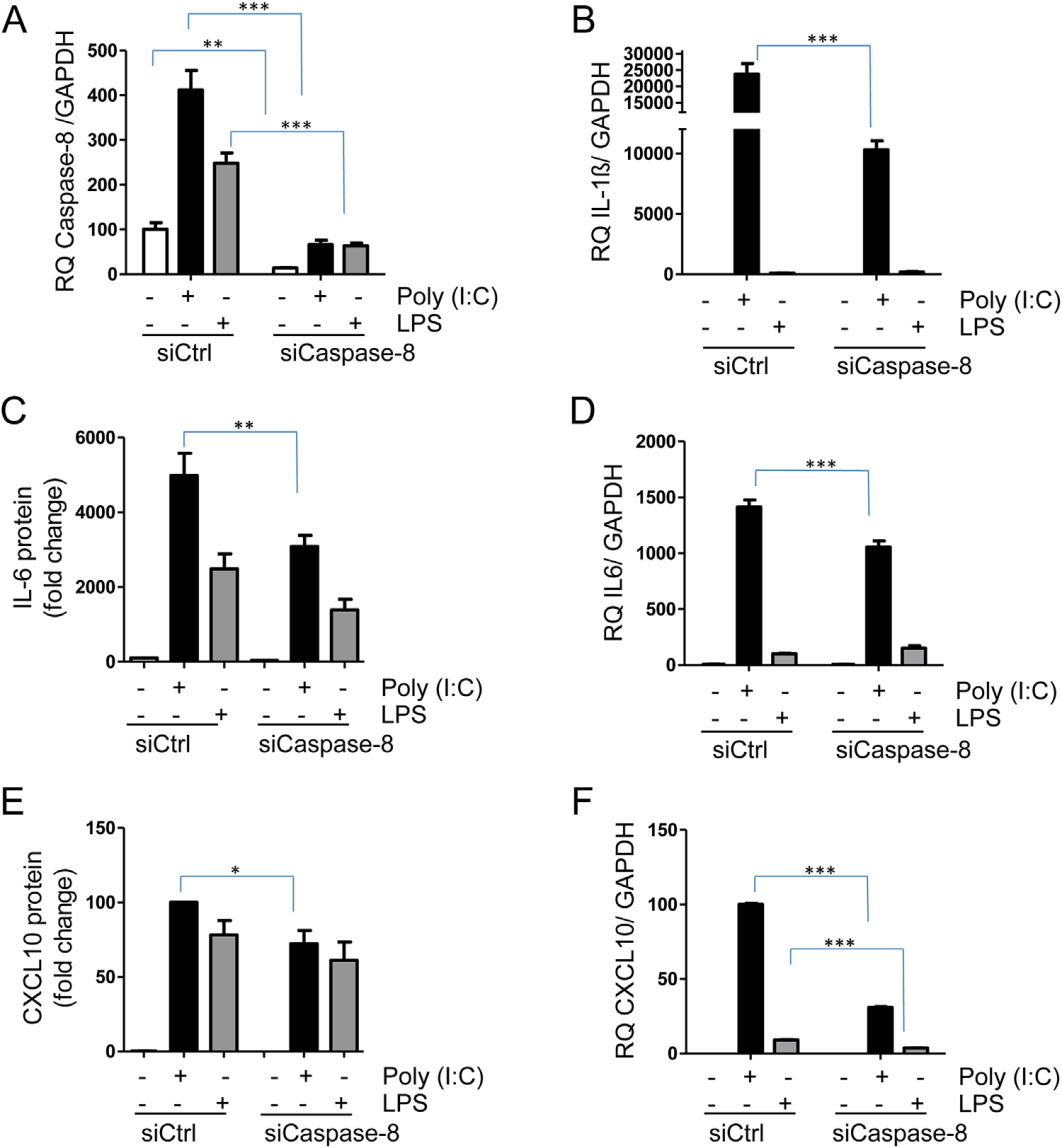
IL‐1β, IL‐6, and CXCL10 expression upon knock down of caspase‐8. hBMSCs were transfected with non‐coding siRNA (siCtrl) or siRNA against caspase‐8 (siCaspase‐8). Forty‐eight hour after transfection the hBMSCs were treated with poly(I:C) (5 µg/ml) or LPS (1 µg/ml) for another 24 h. (A) Caspase‐8 knock‐down at the mRNA level was confirmed by QPCR. (B) IL‐1β mRNA were assessed by QPCR. (C) IL‐6 and (E) CXCL10 protein secretion from hBMSCs was measured by ELISA and are presented as mean + SEM of relative protein concentrations (*n* = 5 independent experiments using hBMSCs obtained from three donors). IL‐6 concentrations in LPS treated cells varied from 3500 to 15000 pg/ml between experiments, and CXCL10 concentrations in LPS treated stimulated cells varied from 600 to 6000 pg/ml between experiments. (D) IL‐6 mRNA and (F) CXCL10 mRNA were assessed by QPCR and data are presented as mean + SD of triplicates from one representative experiment ouf of 3–5 using hBMSCs obtained from three donors. *P < 0.05, **P < 0.01, ***P ≤ 0.001 (Two‐way ANOVA/Bonferroni post test).

Previous studies have suggested an important role for caspase‐8 in TLR‐induced cytokine production in macrophages 20. To dissect a role for caspase‐8 in TLR signaling in hBMSCs, we knocked down caspase‐8 with siRNA. Caspase‐8 mRNA is increased in hBMSCs after stimulation with poly(I:C) and LPS (Fig. 3A). Knocking down caspase‐8 mRNA by approximately 90% (Fig. 3A) led to a reduction of poly(I:C)—induced IL‐1β mRNA by nearly 60% (Fig. 3B). It also reduced the expression of IL‐6 protein in response to poly(I:C) (Fig. 3C) and this difference was also evident at the mRNA level (Fig. 3D). Poly(I:C)‐induced CXCL10 protein expression was slightly but significantly reduced when caspase‐8 was knocked‐down (Fig. 3E), and the reduction was even more evident at the mRNA level (Fig. 3F). Similarly, when we used a caspase‐8 inhibitor, z‐IEDT‐fmk, CXCL10 protein secretion in response to poly(I:C) and LPS was reduced (Supplementary Fig. S1). Hence, IL‐6, IL‐1β, and CXCL10 expression in hBMSCs in response to TLR3 and TLR4 activation is reduced when caspase‐8 is knocked‐down.

### Caspase‐8 mediates NFκB‐signaling

**Figure 4:**
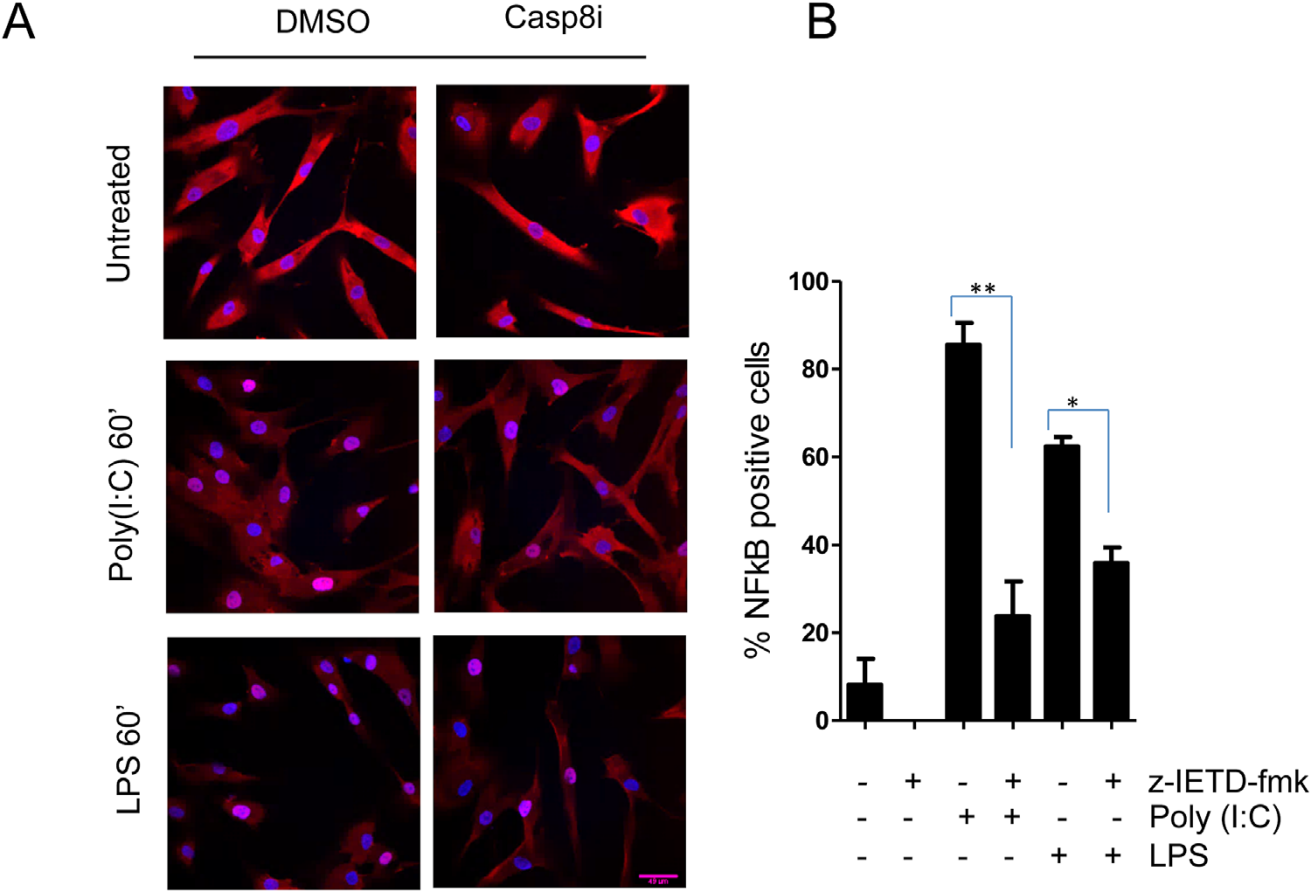
Caspase‐8 mediates NFκB activation. hBMSCs were pre‐treated with caspase‐8 inhibitor z‐IETD‐fmk for 2 h before adding poly(I:C) (5 µg/ml) and LPS (1 µg/ml) as indicated. The cells were stained for NFκB p65, and percent cells with nuclear translocation of NFκB p65 was evaluated by confocal microscopy. Data presented in B is obtained from three independent experiments using hBMSCs obtained from three donors. *P < 0.05, **P < 0.01 (pared Student *t*‐test). Scale bare 49 µm. Original magnification ×400.

TLR‐TRIF signaling can activate both the transcription factors NFκB‐ and IRF3 which promote the induction of inflammatory cytokines. To determine the role of caspase‐8 in mediating the activation of NFκB in hBMSCs, we treated the cells with the z‐IETD‐fmk inhibitor and quantified nuclear translocation of NF‐kB subunit p65 by confocal microscopy as a read‐out for NFκB‐signaling. Both poly(I:C) and LPS treatment promoted p65 nuclear translocation in hBMSCs, which was reduced when the cells were pre‐treated with z‐IETD‐fmk (Fig. 4). Hence, caspase‐8 seems to be important for NFκB signaling.

### Anti‐inflammatory cytokines HGF and TGFβ are increased when caspase‐8 is inhibited

**Figure 5:**
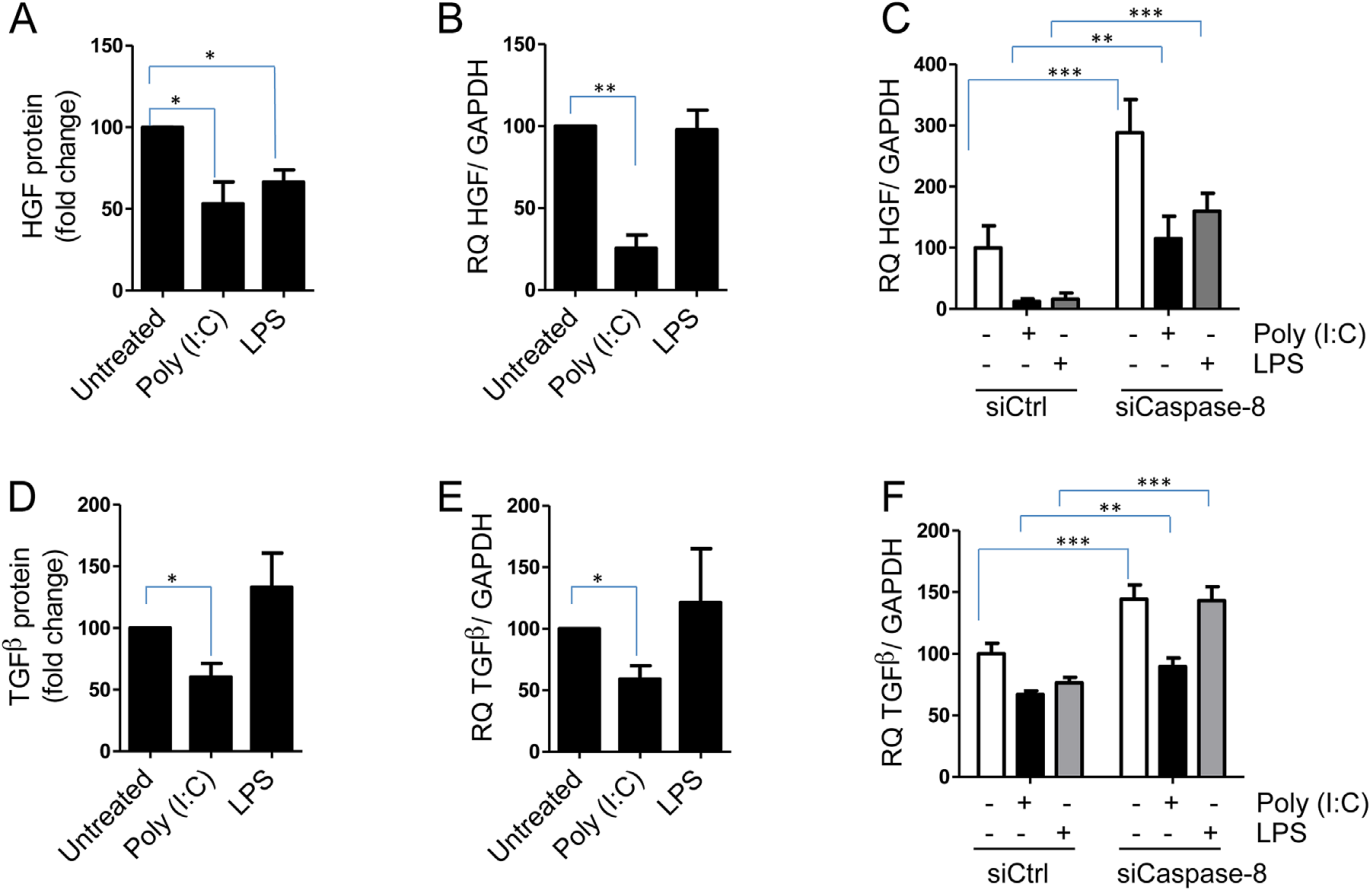
Anti‐inflammatory factors HGF and TGFβ are dowregulated. hBMSCs were treated with poly(I:C) (5 µg/ml) and LPS (1 µg/ml) for 24 h before collection of conditioned media and cells. (A and B) HGF protein and mRNA and (D and E) TGFβ protein and mRNA were quantified by ELISA and QPCR, respectively. hBMSCs were transfected with non‐coding siRNA (siCtrl) or siRNA against caspase‐8 (siCaspase‐8). Forty‐eight hours after transfection the hBMSCs were left untreated or treated with poly(I:C) (5 µg/ml) or LPS (1 µg/ml) for another 24 h. (C) HGF and (F) TGFβ mRNA levels were assessed by QPCR. Data are plotted as mean + SEM (*n* = 3–5 independent experiments) except for (C and F) in which data are plotted as mean + SD of triplicates from one representative experiment out of three. Experiments were performed on hBMSCs from three donors (A, C, D, and F) or four donors (B and E). In (A and D) protein concentrations relative to untreated cells are shown. HGF concentrations in media from untreated cells varied from 100 to 400 pg/ml between experiments and TGFβ concentrations varied from 70 to 250 pg/ml. *P < 0.05, **P < 0.01, ***P ≤ 0.001 determined by pared Student *t*‐test (A, B, D, and E) and Two‐way ANOVA/Bonferroni post test (C and F).

Having shown that caspase‐8 mediated production of the pro‐inflammatory cytokine IL‐1β, CXCL10, and IL‐6 we investigated whether caspase‐8 influenced production of the anti‐inflammatory cytokines HGF and TGFβ. Importantly, poly(I:C) significantly reduced HGF and TGFβ at the protein and mRNA level (Fig. 5A, B, D, and E). LPS, however, had less effect on expression of HGF and TGFβ: it reduced HGF protein, but not HGF mRNA (Fig. 5A and B) and did not influence TGFβ expression, neither at protein or mRNA level (Fig. 5D and E). Strikingly, knocking down caspase‐8 by siRNA increased HGF and TGFβ mRNA in poly(I:C) and LPS treated cells (Fig. 5C and F, respectively). In conclusion, inhibiting caspase‐8 promotes HGF and TGFβ expression.

### Knock‐down of caspase‐8 did not influence cell survival

**Figure 6:**
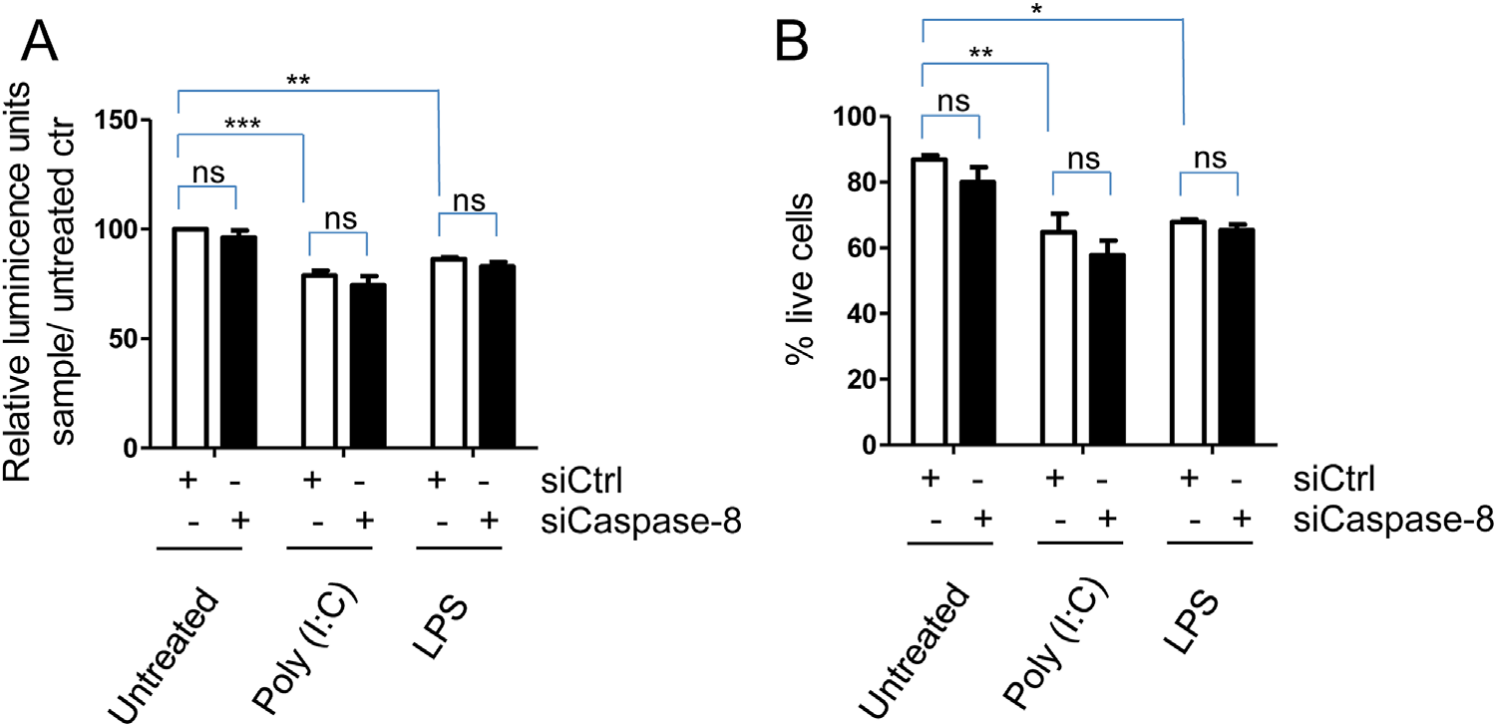
Caspase‐8 does not mediate cell death induced by poly(I:C) and LPS. hBMSCs were transfected with non‐silencing RNA (siCtrl) or siRNA against caspase‐8 (siCaspase‐8). Forty‐eight hours after transfection the hBMSCs were treated with poly(I:C) or LPS for 24 h. (A) Metabolic activity was quantified using the CellTiter glo assay. (B) Cell viability was evaluated by annexin‐V FITC and propidium iodide staining. Data are plotted as mean + SEM of 3–5 independent experiments using hBMSCs obtained from three donors. Untreated siRNA transfected cells was used as a reference. *P < 0.05, **P < 0.01, ***P ≤ 0.001 (Two‐way ANOVA/ Bonferroni post test).

Caspase‐8 was first identified as a pro‐apoptotic caspase, but can also prevent receptor‐interacting serine‐threonine kinase 3 (RIPK3)‐dependent necrosis 31, 32, 33. Hence, we investigated the role of caspase‐8 in hBMSC survival. Poly(I:C) and LPS slightly but significantly reduced viability as evaluated by amount ATP present in the cells (Fig. 6A). Importantly, knocking down caspase‐8 by siRNA did not influence the survival of the hBMSCs per se, nor did it influence poly(I:C) or LPS‐induced cell death (Fig. 6A). Further, when we assessed cell survival by annexin‐PI staining we similarly found that poly(I:C) and LPS reduced the number of live cells, and that caspase‐8 knock‐down did not influence the effect of poly(I:C) and LPS on cell death (Fig. 6B). In conclusion, cell death induced by poly(I:C) and LPS was not mediated by caspase‐8.

## Discussion

In this paper, we demonstrate that caspase‐8 downstream of TLRs regulates cytokine production from hBMSCs. The induction of pro‐inflammatory cytokines, such as IL‐1β, IL‐6, and CXCL10 and the inhibition of anti‐inflammatory HGF and TGFβ in hBMSCs were, at least partly, dependent on caspase‐8. In contrast, silencing of caspase‐8 did not influence cell survival. Our results point to a role for caspase‐8 in regulating the immune modulating phenotype of hBMSCs.

Caspase‐8, which was initially identified as an initiator caspase in death receptor induced apoptosis, has recently been shown to have an important function in modulating inflammation 33. For example, caspase‐8 can proteolytically cleave pro‐IL‐1β in response to LPS and poly(I:C) stimulation 34, 35, 36. In another study caspase‐8 was shown to be important for TLR‐induced inflammasome priming, and for the production of pro‐inflammatory cytokines 37. Here we demonstrate that in hBMSCs, caspase‐8 is not only important for the induction of pro‐inflammatory cytokines, but also for the reduction of TGFβ and HGF secretion in response to poly(I:C) and LPS. TGFβ and HGF are key factors in terms of mediating the immune‐dampening effect of hBMSCs. In particular, both cytokines can suppress the maturation of dendritic cells and stimulate the generation of regulatory T‐cells 8, 38, 39, 40. Thus, exposure of hBMSCs to TLR agonists is likely to reduce the immune suppressive potential of the hBMSCs in a caspase‐8‐dependent manner. However, to conclude on this matter careful examination of how inhibiting or knocking down caspase‐8 in hBMSCs may impact on immune cell function in vitro and in vivo is needed. In this respect it is interesting to note that a recent paper demonstrated that knocking down caspase‐8 in hBMSCs upregulated HGF, among other cytokines, and improved the therapeutic potential of the hBMSCs in the treatment of infarcted hearts 41.

We found that the induction of the pro‐inflammatory cytokines IL‐1β, IL‐6, and CXCL10 in hBMSCs was largely dependent on a TRIF‐dependent pathway upstream of caspase‐8, as knocking down TLR3, TRIF, and caspase‐8 resulted in reduction of IL‐1β, CXCL10, and IL‐6. This is in line with previous studies on mouse macrophages, showing that TRIF, rather than MYD88‐dependent pathways promoted caspase‐8 activation and IL‐1β production 35, 36. Notably, we found that TLR3‐ and TRIF knockdown resulted in greater reduction of both IL‐1β, CXCL10, and IL‐6 compared with caspase‐8 knockdown, suggesting that caspase‐8‐independent pathway(s) downstream of TLR‐TRIF contribute, or that low levels of caspase‐8 are sufficient to maintain cytokine production. Our data, further suggest that the reduction of TGFβ and HGF is mediated by another signaling pathway than TLR‐TRIF, since expression of these cytokines was not influenced when TLR3 or TRIF were knocked‐down (data not shown). Identifying the receptor/signaling pathway mediating this effect is of great interest and should be addressed in future studies. Candidate receptors mediating the effect on poly(I:C) on HGF and TGFβ are the cytosolic receptors RIG‐I or MDA5. Importantly, caspase‐8 has been shown to mediate signaling downstream also of these receptors 42.

Caspase‐8 has in several studies been shown to mediate NFkB‐signaling 19, 20, 23, but how this happens at the molecular level is still unclear. Some studies found that enzymatic activity of the caspase was important 35, while others found that the non‐apoptotic activity of caspase‐8 was independent of its enzymatic activity 21, 22. Our results support the notion that the enzymatic activity of caspase‐8 is important, since the inhibitor z‐IETD‐fmk, which is a cell permeable peptide which binds irreversibly to the catalytic site of caspase‐8, reduced NFkB nuclear translocation (Fig. 4) and reduced CXCL10 production (Supplementary Fig. S1). In addition to the NFkB‐pathway, the interferon regulatory protein (IRF) pathway 30 is also activated in hBMSCs upon TLR stimulation. Previous reports showed an inhibitory effect of caspase‐8 on IRF3‐signaling downstream RIG‐I 43, 44. If and how caspase‐8 modulates the IRF‐pathway in hBMSCs was not addressed in this study. Thus, further studies are needed to conclude on the importance of caspase‐8 for IRF signaling in response to TLR agonists in hBMSCs.

Activation of TLR3‐ and 4‐ can induce cell death via RIP3‐dependent pathway(s) 26, 45. Untransfected hBMSCs, however, tolerated quite high concentraions of poly(I:C) and LPS, as we observed only about 5% cell death when the cells were treated for 24 h or more with poly(I:C) at 5 µg/ml (data not shown). Transfected hBMSCs were more fragile, and the fraction of dead cells increased to about 20% when they were exposed to poly(I:C) and LPS for 24 h (Fig. 6). Importantly, knocking down caspase‐8 did not influence cell survival, neither in untreated nor in poly(I:C) or LPS treated cells. Thus, although activated caspase‐8 can act as an inhibitor of necroptosis 33, in TLR agonist treated‐hBMSCs caspase‐8 might be more important for regulating cytokine production.

hBMSCs have great potential in stem cell therapy because of their ability to differentiate and their immunosuppressive properties. It is therefore important to understand how these cells respond to inflammatory stimuli. Our data suggest that caspase‐8 can modulate hBMSCs into a pro‐inflammatory phenotype by simultanously increasing pro‐inflammatory cytokines and impairing anti‐inflammatory factors (Supplementary Fig. S2). Hence, targeting caspase‐8 might be beneficial when hBMSCs are used in treatment of inflammatory disorders.

## Materials and Methods

### Cells and culture conditions

Human bone marrow‐derived mesenchymal stromal cells from eight different healthy male donors (MSC, Lonza, Walkersville, Maryland, donors lot #; 1F3422, 318006, 1F4287, 307219, 374385, 351482, 422610, 8F3520) were cultured in mesenchymal stem cell growth media (MSCGM) (Lonza) at 37°C in a humidified atmosphere containing 5% CO_2_ according to the manufacturer's instructions. The cells were passaged for a maximum of seven times. For experiments, 70 000 hBMSCs were seeded per cm^2^. At 80% confluency the cells were treated as indicated in the figure legends with TLR2/TLR1 agonist Pam_3_Cys‐Ser‐Lys_4_ (Pam_3_CSK_4_) (1 μg/ml) (EMC microcollections GmbH, Tuebingen, Germany), the TLR3 agonist polyinosinic–polycytidylic acid (poly(I:C)) (5 μg/ml) (Amersham Life Science, Little Chalfont, United Kingdom), TLR4 agonist lipopolysaccharide (LPS) obtained from *E. coli* O111:B4 (LPS B4) (1 μg/ml) (InvivoGen, SanDiego, California) and ultrapure preparations of LPS from *E. coli* K12 (LPS K12) (1 μg/ml) (InvivoGen). LPS B4 was used in generating the data in Supplementary Table S1, LPS K12 was used in all other experiements. Caspase‐8 Inhibitor z‐IETD‐fmk (R&D Systems, Minneapolis, Minnesota) was added to the cells 2 h before cells were treated with TLR agonists.

### Cytokine measurements

Cells were seeded and treated for 24 h with TLR agonists as described above. The culture media was harvested and cytokine concentrations measured using Bio‐Plex Pro human cytokine 27‐plex assay (Bio‐Rad Laboratories, Hercules, California) according to the manufacturer's instructions. IL‐6, CXCL10, TGFβ, and HGF were quantified using Duo‐set ELISAs (R&D Systems, Abingdon, UK) following the manufacturer's instructions.

### Quantitative transcription polymerase chain reaction (qRT‐PCR)

Total RNA was isolated using High Pure RNA Isolation Kit (Roche, Mannheim, Germany). Complementary DNA (cDNA) was synthesized from total RNA using the High Capacity RNA‐to‐cDNA kit (Applied Biosystems, Carlsbad, California). PCR was performed using StepOne Real‐Time PCR System and Taqman Gene Expression Assays (Applied Biosystems) using standard settings (2′ 50°C, 10 ′ 95°C, 40 cycles at 95°C for 15 sec, 1′ 60°C). The comparative Ct method was used to estimate relative changes in gene expression using GAPDH as housekeeping gene. The following primers from Thermo Fischer Scientific Inc., Waltham, Massachusetts. were used: GAPDH (Hs99999905_m1), IL‐1β (Hs01555410_m1), IL‐6 (Hs00985639_m1), CXCL10 (Hs00171042_m1), TLR3 (Hs01551077_m1), Caspase‐8 (Hs01018151_m1), TRIF (TICAM1, Hs01090712_m1), TGF β (Hs00998133_m1), and HGF (Hs00300159_m1). The analyses were carried out using the Applied Step One software 2.1 (Applied Biosystems, Carlsbad, California).

### Cell viability

Cell viability was determined by flow cytometry using annexin V‐FITC and propidium iodide (PI) (APOTEST‐FITC kit, Nexins Research, Hoeven, Netherlands). Cells were treated as indicated in the figure legends before incubation with annexin V‐FITC on ice for 1 h. Propidium iodide (1.4 µg/ml) was added 5 min before cells were analyzed. Data were collected using FACS LSRII and analyzed by BD FACS Diva™ Software (Becton Dickinson, Franklin Lakes, New Jersey) and FlowJo (Tree Star Inc. Ashland, Oregon). Cell viability was also determined using the Cell Titer‐Glo assay (Promega, Madison, Wisconsin). Cells were seeded in 96 well optical plates and treated as indicated in the figure legends before Cell Titer‐Glo reagent was added following the manufacturer's instructions. Luminescence was determined using Victor 1420 multilabel counter (PerkinElmer Inc., Waltham, Massachusetts).

### Short interfering RNA‐transfection

BMSCs were grown to 80% confluency and transfected with 5 nmol siRNA using Lipofectamine RNAiMAX transfection reagent following the manufacturer's instruction. 48 h after transfection the cells were treated with poly(I:C) (5 μg/ml) or LPS K12 (1 μg/ml) as described in the figure legends. Hs_TLR3_5 (Cat #SI000050043), was used to target TLR3, and Hs_Caspase 8_7 (Cat #SI00299593) and Caspase 8_11 (Cat #SI02661946) were used to target caspase‐8 (Qiagen, Crawley, United Kingdom) and siTICAM (#AM16708, Life Technologies, Carlsbad, California) was used to target TRIF. Negative control siRNA (Cat #1027310, Qiagen) was used as non‐silencing control RNA.

### Western blot

Cells were treated with TLR‐agonists and siRNA as indicated in the figure legends. Then the cells were washed twice in PBS before they were lysed in lysis buffer (50 mM Tris–HCl, 1% NP40, 150 mM NaCl, 10% glycerol, 1 mM Na_3_VO_4_, 50 mM NaF and Complete protease inhibitor [Roche Diagnostics, Mannheim, Germany]). The samples were denatured in 1× NuPage LDS sample buffer supplemented with 25 mM DTT for 10 min at 70°C before they were separated on 10% Bis‐Tris polyacrylamide gel and transferred to a nitrocellulose membrane using the iBlot Dry Blotting System (Invitrogen, Camarillo, California). The membrane was blocked using 5% bovine serum albumin (Sigma–Aldrich, St. Louis, MO) in Tris‐buffered saline with 0,1% Tween. TLR3‐ and pro IL‐1β antibodies (Cell Signaling Technology, Beverly, Missouri) were used for protein detection. Monoclonal anti‐GAPDH (Abcam, Cambridge, United Kingdom) was used as loading control. For visualization the blots were incubated with horseradish peroxidase (HRP) conjugated immunoglobulin's (DAKO, Glostrup, Denmark) and developed with Super Signal West Femto Maximum Sensitivity Substrate (Thermo Scientific, Rockford, Illinois). Images were obtained with LI‐COR Odyssey Fc and analyzed using Image Studio Software (LI‐COR, Lincoln, Nebraska).

### Confocal microscopy

Nuclear translocation of p65 in response to poly(I:C) or LPS in the presence or absence of the caspase‐8 inhibitor z‐IETD‐fmk was assessed by confocoal microscopy. hBMSCs (2000 cells/well) were seeded in 96 well glass bottom plates and treated with 100 µM Caspase‐8 inhibitor z‐IETD‐fmk for 2 h before adding poly(I:C) and LPS as indicated in the figure legend. Then, the cells were fixed with 4% paraformaldehyde (PFA) and permeablized with 0,1% Triton X‐100. Blocking was performed with 10% BSA in PBS containing 0.1% Tween 20 (PBST) for 1 h, followed by rabbit anti‐p65 antibodies (#8242, Cell Signaling) diluted 1:100 in 1% BSA in PBST overnight at 4°C. The cells were stained with Alexa‐647 conjugated goat anti‐rabbit for 45 min before nuclei were stained with Hoechst 33342 (Life Technologies). Cells in which p65 was localized in the nucleus were counted using a Zeiss LSM 510 confocal microscope (40×).

### Statistical analyses

Statistical analyses were performed using Prism 5.0. Differences between two groups were determined by the Student's paired two‐tailed *t*‐test. For comparisons of three or more groups two‐way ANOVA with Bonferroni post test was performed. *P* values less than or equal to 0.05 were considered significant.

## Conflict of Interest

The authors declare no commercial or financial conflict of interest.

## Supporting information

Additional supporting information may be found in the online version of this article at the publisher's web‐site.

**Table S1**. Cytokines produced by BMSCs treated with TLR agonists for 24 h.

**Figure S1**. Inhibiting caspase‐8 reduces CXCL10 expression.
**Figure S2**. Graphical summary.
