# Supplementary material for: Caspase‐8 regulates the expression of pro‐ and anti‐inflammatory cytokines in human bone marrow‐derived mesenchymal stromal cells
Source: Immun Inflamm Dis. 2016 Jul 21;4(3):327–37. doi: 10.1002/iid3.117 (PMC5004287; doi:10.1002/iid3.117)
Supplement: Supplementary file 1 — Table S1. Cytokines produced by BMSCs treated with TLR agonists for 24 h. [file IID3-4-327-s002.docx]

**Supplementary Table 1. Cytokines produced by BMSCs treated with TLR agonists for 24 hours.**

|  | **Untreated** | | **P3CSK4 (1 μg/ml)** | | **Poly(I:C) (5 μg/ml)** | | **LPS (1 μg/ml)** | |
| --- | --- | --- | --- | --- | --- | --- | --- | --- |
|  | *Donor 1* | *Donor 2* | *Donor 1* | *Donor 2* | *Donor 1* | *Donor 2* | *Donor 1* | *Donor 2* |
| **IL-1β** | - | - | - | - | 9 | 10 | - | - |
| **IL-1ra** | 19 | - | 33 | - | 141 | 145 | 40 | - |
| **IL-6** | 1737 | 1265 | 2774 | 1071 | 20536 | 20536 | 6600 | 3043 |
| **IL-8** | 630 | 352 | 1571 | 178 | 16499 | 11839 | 6036 | 2670 |
| **IL-9** | 15 | - | 22 | - | 57 | 61 | 23 | 13 |
| **IL-17** | - | - | - | - | 34 | 40 | - | - |
| **Eotaxin** | 48 | 27 | 69 | 22 | 211 | 216 | 71 | 45 |
| **Basic FGF** | - | - | 5 | - | 10 | 18 | 8 | - |
| **G-CSF** | - | - | 13 | - | 1465 | 3280 | 16 | - |
| **GM-CSF** | - | - | - | - | 12 | 22 | - | - |
| **IFN-γ** | 251 | 152 | 282 | 147 | 547 | 769 | 293 | 244 |
| **CXCL10** | 42 | 11 | 54 | 12 | 32446 | 32446 | 65 | 31 |
| **CCL2** | 279 | 134 | 290 | 89 | 1268 | 1454 | 573 | 336 |
| **CCL3** | - | - | - | - | 435 | 348 | - | - |
| **CCL4** | - | - | - | - | 494 | 383 | - | - |
| **CCL5** | 2 | - | 4 | - | 1015 | 5273 | 5 | 2 |
| **TNF-α** | 7 | - | 10 | - | 43 | 64 | 12 | - |
| **VEGF** | 3885 | 1995 | 5965 | 1254 | 7158 | 3562 | 5900 | 2574 |
| **IL-7** | 14 | 15 | 11 | 13 | - | - | 14 | 12 |
| **IL-10** | 21 | 30 | 19 | 25 | 14 | 12 | 19 | 17 |
| **IL-12 (p70)** | 201 | 285 | 213 | 261 | 123 | 97 | 187 | 168 |
| **IL-13** | 14 | 20 | 15 | 17 | 11 | 10 | 15 | 16 |
| *Not detected: IL-2, IL-4, IL-5, IL-15 and PDGF-BB. Cytokine concentrations in pg/ml.* | | | | | | | | |
