# Supplementary material for: Caspase‐8 regulates the expression of pro‐ and anti‐inflammatory cytokines in human bone marrow‐derived mesenchymal stromal cells
Source: Immun Inflamm Dis. 2016 Jul 21;4(3):327–37. doi: 10.1002/iid3.117 (PMC5004287; doi:10.1002/iid3.117)
Supplement: Supplementary file 2 — Figure S1. Inhibiting caspase‐8 reduces CXCL10 expression. Figure S2. Graphical summary. [file IID3-4-327-s001.docx]

**Supplementary Figure 1. Inhibiting caspase-8 reduces CXCL10 expression.** BMSCs were treated with DMSO control or caspase-8 inhibitor z-IETD-fmk before adding (A) poly(I:C) (5 μg/ml) and (B) LPS (1 μg/ml) for 24 hours. CXCL10 protein levels were measured by ELISA. Data are presented as mean + SEM (n=5) relative to poly(I:C) (A) or LPS (B) control siRNA treated cells. CXCL10 concentrations in media from poly(I:C) treated cells varied from 1-8 ng/ml and for LPS treated cells from 0.4 -1 ng/ml between experiments. ***P <0.001 (Two-way ANOVA/Bonferroni post test).

**Supplementary Figure 2. Graphical summary.** LPS and poly(I:C) induce pro-inflammatory cytokines from hBMSCs via TLR -TRIF signaling. LPS and poly(I:C) inhibit HGF and TGFβ secretion from MSCs. Caspase-8 promotes IL-1β, IL-6 and CXCL10 and inhibits HGF and TGFβ secretion. Hence, inhibiting caspase-8 may promote an immunosuppressive MSC phenotype.
